# A Joint Prosodic Origin of Language and Music

**DOI:** 10.3389/fpsyg.2017.01894

**Published:** 2017-10-30

**Authors:** Steven Brown

**Affiliations:** Department of Psychology, Neuroscience & Behaviour, McMaster University, Hamilton, ON, Canada

**Keywords:** language, music, speech, song, evolution, prosody, intonation, emotion

## Abstract

Vocal theories of the origin of language rarely make a case for the precursor functions that underlay the evolution of speech. The vocal expression of emotion is unquestionably the best candidate for such a precursor, although most evolutionary models of both language and speech ignore emotion and prosody altogether. I present here a model for a joint prosodic precursor of language and music in which ritualized group-level vocalizations served as the ancestral state. This precursor combined not only affective and intonational aspects of prosody, but also holistic and combinatorial mechanisms of phrase generation. From this common stage, there was a bifurcation to form language and music as separate, though homologous, specializations. This separation of language and music was accompanied by their (re)unification in songs with words.

Theories of the origins of language generally fall into the two broad categories of vocal and gestural models (Corballis, 2002; MacNeilage and Davis, 2005; Armstrong and Wilcox, 2007; Arbib, 2012; McGinn, 2015). Given that humans have evolved species-specific capacities for both vocal imitation and gestural imitation (Donald, 1991), a central question is whether language evolved initially as a system of vocalization or one of gesture, since imitative mechanisms are critical to evolutionary accounts of language acquisition. Gestural theories of language have grown in popularity in recent years due to their association with mirror-neuron-based models of action observation (Arbib, 2012). However, vocal theories have a far deeper grounding in historical models of language, going back to the ancient Greeks. During the Renaissance, not only was it commonplace to talk about the evolutionary connection between language and music, but both functions were seen as being clearly rooted in the vocal expression of emotion (Condillac, 1746; Rousseau, 1781; Thomas, 1995), a trend that continued into Darwin's day (Spencer, 1857, 1890; Darwin, 1871, 1872) and through to the early twentieth century (Wallaschek, 1891; Newman, 1905; Nadel, 1930; Sachs, 1943).

While contemporary vocal accounts of language origin do not deny the linkage between speech and emotion, they do not consider it to be central to their models, focusing instead on the articulatory innovations of speech—such as complex phonemic repertoires, the nature of syllable structure, vocal learning, descent of the human larynx, among others—or the origins of symbolization *per se*, separate from emotional communication. Some models talk about the origins of speech in “singing” (Darwin, 1871; Jespersen, 1922), but there are problems associated with this invocation of singing. Singing as a human behavior implies something musical, but the musicality of the posited ancestral singing mechanism is not specified. Singing simply becomes a counter-state to speaking (i.e., language-based vocalizing), rather than being something truly musical, as predicated on the tonal principles of scale structure. When Jespersen (1922) claimed that our ancestors “sang out their feelings long before they were able to speak their thoughts” (p. 436), his notion of singing included such diverse vocalizations as the singing of birds, the roaring of mammals, and the crying of babies. Likewise, Fitch (2010) referred to music as being an example of “bare phonology,” viewing music as basically a counter-state to propositional speech. The aim of this essay is to propose a joint prosodic model of the origins of language and music, but to avoid the pitfalls of talking about a singing or phonological mechanism that has no musical specifications. As with my earlier writings on the topic (Brown, 2000a,b, 2007), my focus here will be on phylogenetic issues of cognitive structure, rather than on Darwinian issues of adaptiveness and selection mechanisms (which I have discussed in detail in Brown, 2000b).

While vocal and gestural models have generally been placed in opposition to one another, it is far more reasonable instead to see vocalization and gesture as complementary communicative specializations (McNeill, 2005; Arboitiz and Garcia, 2009; Arbib, 2012; Arboitiz, 2012; Garcia et al., 2014), as suggested in Figure 1. Gesture seems particularly well-suited to iconically convey information about the spatial properties of objects and actions through pantomimic gestures (Armstrong and Wilcox, 2007), which the vocal system cannot easily achieve through iconic means. By contrast, vocal prosody seems better suited to convey information about the emotional meaning of a perceived object for the communicator, in other words its consequentiality. To my mind, the co-speech gestures of modern speech are essentially pantomimes (Beattie, 2016), and might therefore comprise “fossils” of an early gestural stage of language evolution that was pantomimic. Another potential fossil consists of what I will call “acoustic pantomimes,” namely iconic sounds, such as onomatopoeias. Such pantomimes are able to represent the sound-generating properties of objects and actions—as in the “ruff” of a dog barking—as well as non-vocal object-properties like size, height, velocity, and temperature that are correlated with the acoustics of objects (Nygaard et al., 2009; Dingemanse et al., 2015, 2016; Perlman et al., 2015; Svantesson, 2017). Figure 1 suggests that pantomimes in both the vocal and gestural domains served as parallel precursor stages on the road to symbolization for each route of communication.

**Figure 1:**
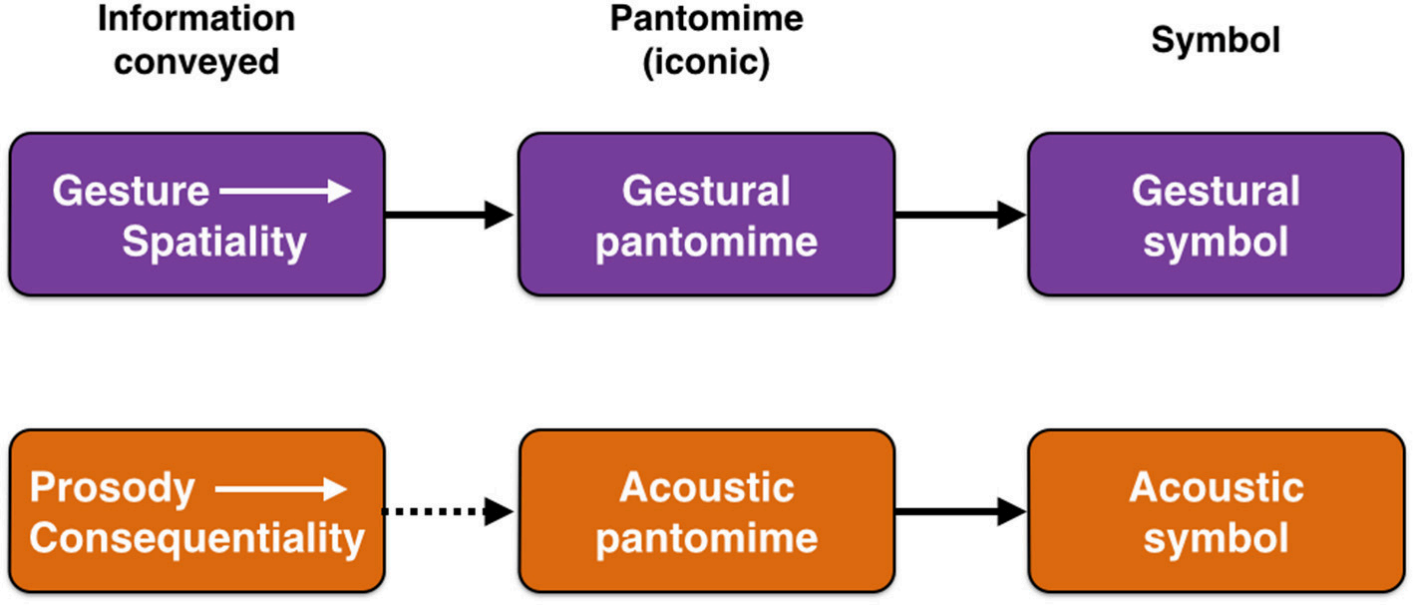
Prosody and gesture compared. The figure shows parallel gestural and vocal routes to the origin of language by means of either gestural symbols (sign language) or acoustic symbols (speech). The pantomime stage in the middle of both routes is one of iconic representation. The term “acoustic pantomime” refers to iconic words (onomatopoieas) and so-called sound symbolisms. The dashed arrow in the second row suggests that, compared to the more natural relationship between gesture's spatiality and visual pantomimes, the connection between prosody's conveyance of consequentiality and acoustic pantomimes is more remote.

Beyond the intermediate iconic stage of pantomime, the bulk of the symbolic function of language resides with acoustic symbols, rather than gestural symbols, with the prominent exception of sign languages among the deaf. While I am quite sympathetic to gestural models of language origin, I will put them aside from this point onward in order to examine the basic question raised above, namely whether language and speech arose as an offshoot of the vocal expression of emotion. Most evolutionary speech models ignore prosody altogether and instead focus on the anatomical changes to the human articulatory system that permitted the emergence of syllable structure, including descent of the larynx. An interesting example is MacNeilage's (1998) “frame/content” model, which proposes that mandibular oscillations in great apes (e.g., lip smacking) provided a scaffold upon which syllable structure in speech may have arisen (see also MacNeilage and Davis, 2005; Ghazanfar et al., 2012). What is central to this model is that such oscillations are voiceless in non-human primates, and that the key innovation for speech would be the addition of phonation to such oscillations so as to create alternations between vowels (open vocal tract) and consonants (closed or obstructed vocal tract).

A central tenet of syllabic accounts of language evolution is the notion of “duality of patterning” (Hockett, 1960; Ladd, 2012), which argues that the acoustic symbols of speech—i.e., words—are built up from meaningless constituents. Words are comprised of fundamental units called phonemes, but none of the phonemes themselves have intrinsic meanings (although sound-symbolic accounts of language origin argue that phonemes have non-random occurrences across word classes and hence may have some minor symbolic content; Monaghan et al., 2016). Through the kinds of mandibular-oscillatory mechanisms that MacNeilage's (1998) speech model highlights, phonemes get combined to form syllables, most universally as alternations between consonants and vowels. This process of syllable formation is not merely oscillatory but is *combinatorial* as well. From a small and fixed inventory of consonants and vowels—generally a few dozen in any given language—these phonemes become combined to form syllables. Such syllables may constitute words in and of themselves (“bee”), or they may be combined with other syllables to form polysyllabic words (“being,” “Beatrice”). Finally, through a different type of combinatorial mechanism, words can be combined with one another to form phrases and sentences through compositional syntactic mechanisms, as described below in the section “Syntax evolution and the ‘prosodic scaffold’ ”.

## The prosodic scaffold

I would like to reflect on what is missing in the standard syllabic account of speech and language just presented. Much of it comes down to whether one thinks of language evolution as serving a purely cognitive function for an individual (Berwick, 2011) or instead a communicative function for a group of individuals (Tomasello, 2003; Robinson, 2005; Scott-Phillips, 2017). In a later section of this article about syntax, I will describe this as a contrast between a “monologic” view (focused on internal thought) and a “dialogic” view (focused on social interaction) of language evolution. If one thinks about language and speech as a dialogic system of social communication, as many theorists suggest, then vocal prosody is critically missing from the syllabic account.

Prosody is characterized by a number of expressive melodic and rhythmic features of an utterance that convey information about emotion, intention, attentional focus, and communicative stance (Scherer, 2003). It is quite different from what has been described for syllables. It is not combinatorial, but is instead holistic, conveying emotional meanings according to rules of expression that govern emotional modulations of vocal pitch, loudness, timing/duration, and timbral features, as based on the valence and intensity of the expressed message. An influential cross-species account of this is found in Morton's (1977) set of “motivation-structure rules,” themselves based on thinking going back to Darwin's (1872) treatise on *The Expression of Emotions in Man and Animals*. For example, aggression is conveyed with harsh, low-frequency sounds, whereas fear and submission are conveyed with more tone-like, high-frequency sounds. According to a prosodic account, it is not sufficient to think of “bee” as an arbitrary acoustic symbol for an insect that is generated through the combination of meaningless phonemes. In actual interpersonal communication, “bee” will be vocalized in such a manner as to convey the consequentiality of that insect for the speaker and his/her audience, as governed by prosodic rules of expression that communicate the emotional valence and intensity of that insect for the communicators. In other words, the vocal expression “Bee!!” during interpersonal communication conveys as much about the speaker's emotions and intentions as it does about the object itself. The holistic nature of prosody operates such that the declamation “Bee!” constitutes a complete utterance; it is essentially a one-word sentence.

It is important to consider that prosody is not some add-on to the combinatorial phonemic mechanism for generating syllable strings and sentences, but instead the reverse: it is the foundation of vocal communication, not least speech. Phonemic mechanisms must be superimposed upon *it*; it is not the case that a monotone string of phonemes becomes “melodized” by prosody after the fact. Prosody is intrinsic to the generative mechanism of speech and is in fact the primary consideration in communication, namely the speaker's emotional intent and message. While there is ample evidence for a “prosody first” model of speech planning when applied to the linguistic-prosodic levels of phonological and phonetic encoding (Keating and Shattuck-Hufnagel, 2002; Krivokapic, 2007, 2012), there is still minimal experimental work regarding the generative aspect of the expression of affect in speech. Instead, most language models place “conceptual structure” at the highest level of the communicative hierarchy (Levelt, 1999), implicating the domain of semantics and thus words. What is missing here is an overarching “emotional semantics” of communication in which emotion is a primary component in the production of speech, preceding word selection. Other theorists have made similar claims with reference to “ostensive communication” or the communication of intent (Scott-Phillips, 2017). Consider the sentence “It's a bee.” That same string of words would not only be uttered in a dramatically different manner between seeing a photograph of a bee in a magazine as compared to seeing an actual bee on one's dinner plate, but the behavioral consequences would, in theory, be quite different. So, while linguists are able to support a “prosody first” model when it comes to linguistic prosody vis-à-vis syntax, I would argue that we need to expand this to have the planning of affective prosody occur at an even earlier stage in the process.

Based on this reasoning, I would like to propose a “prosodic scaffold” model in which overall communicative (intentional, emotional) meaning is the primary factor being conveyed in speech and in which the combinatorial and compositional mechanisms of speech's words and utterances act to “fill out” a prosodic scaffold. This scaffold is comprised of (1) affective prosody, which refers to the vocal expression of emotions, usually acting on the utterance at a global level; and (2) linguistic prosody, which refers to a set of both local and global mechanisms for conveying emphasis (stress, prominence, focus), modality (e.g., question vs. statement), among other features (Cruttenden, 1997; Ladd, 2008). Because I am going to apply concepts about linguistic prosody to music in this article, I will avoid confusion in nomenclature by referring to it as “intonational” prosody from this point on.

### Affective prosody

There are affective mechanisms that modulate the overall pitch height, loudness, and tempo of a spoken utterance in order to convey the emotional valence and intensity of the communicator's meaning. For any given sentence, happiness is typically conveyed by high, loud, and fast prosody, while sadness is conveyed by the opposite profile (Banse and Scherer, 1996). The same is true for the expression of these two emotions in music (Juslin and Laukka, 2003). These types of affective prosodies often work in a global fashion, affecting the entire scaffold of the phrase. For example, happiness both moves the scaffold to a higher vocal register and compresses it in time, while sadness moves it to a lower register and expands it in time. In both cases, the holistic formula of a declarative sentence is preserved, but its acoustic properties are modified by emotional intent.

### Intonational prosody

**Figure 2:**
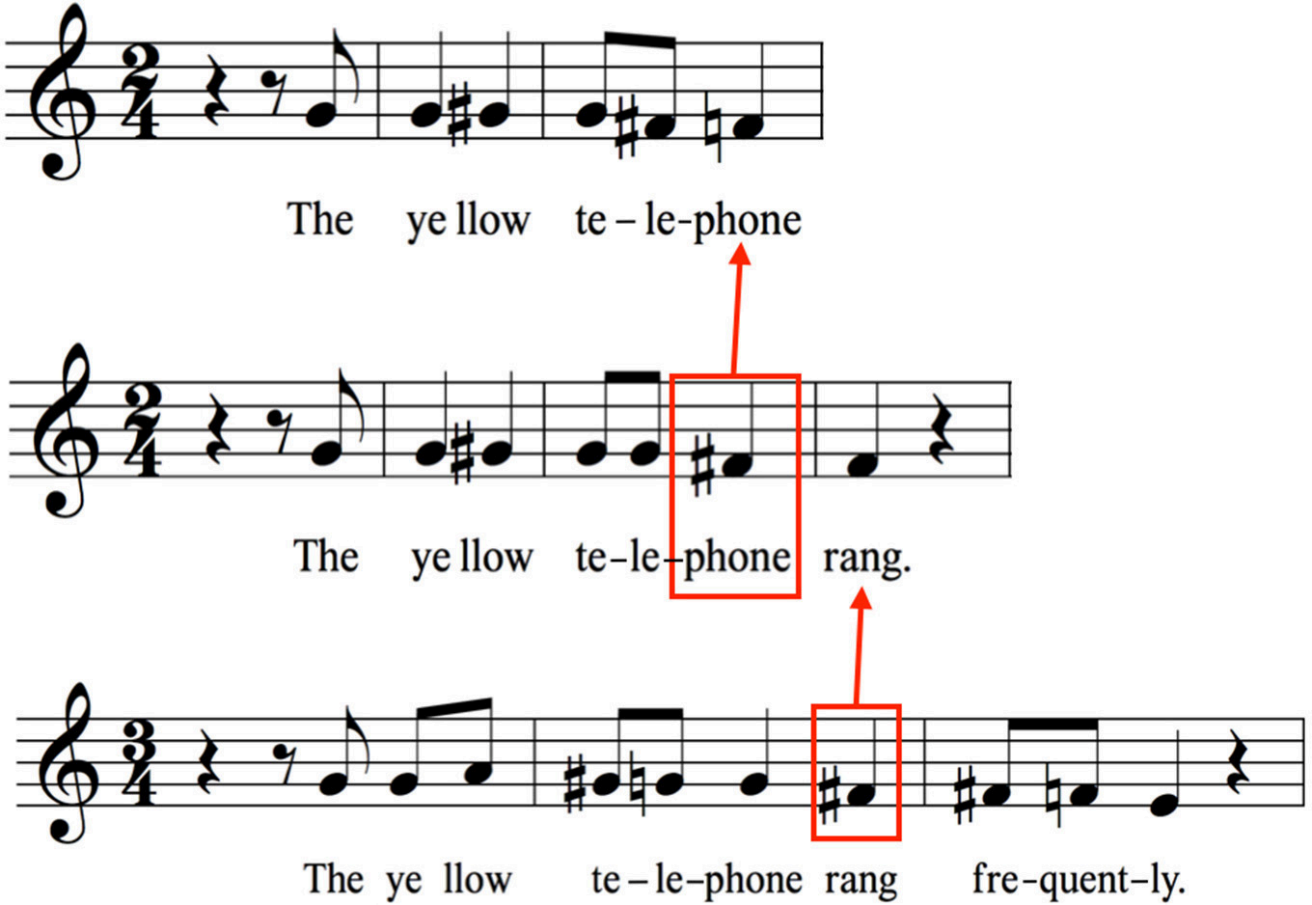
Prosodic scaffolds. The notion of a prosodic scaffold refers to the melodorhythmic shell of a spoken utterance, as based on its prosodic features. Many declarative sentences have simple declining pitch profiles, as shown in musical notation on the top line for the phrase “The yellow telephone.” A lengthening of the utterance (middle line) produces a suspension of the declination found in the shorter phrase, as shown by the red box around “phone,” which is higher in pitch than “phone” in the first phrase. The same mechanism occurs again on the bottom line when the utterance is lengthened a second time. Here the suspension occurs on “rang” (red box), which is higher than “rang” in both preceding sentences. Data are taken from Chow and Brown (submitted), based on the relative-pitch productions of 19 native speakers of English. The relative pitch is shown on a treble clef for convenience of visualization. The absolute pitches are about an octave lower.

A majority of spoken utterances are declarative and are characterized by stereotyped intonations having either arched or descending pitch contours (Halliday, 1967; Cruttenden, 1997). This is exemplified in the top row of Figure 2 for a single sentence using musical notation, which is taken from Chow and Brown's (submitted) analysis of the relative-pitch profiles of spoken sentences, as averaged across a group of 19 speakers. One can readily observe the basic pattern of declination (i.e., falling pitch) characteristic of declarative sentences. The holistic nature of this formula is shown by the fact that, when the sentence is lengthened through the addition of words at the end, the declination process is *suspended* until a later point in the sentence. What this indicates is that the holistic prosodic scaffold of a declarative sentence, with its descending contour, is “filled out” in the longer sentence by suspending the point of declination until the terminal word.

If we contend that the vocal expression of emotion was the precursor to speech, then the evolution of the phonemic combinatorial mechanism had to find a way to create words (strings of segmental units) and phrases in the context of communicating emotional meanings by filling out a prosodic scaffold. The alternative idea, namely that prosody is some type of *post-hoc* affective modulation of a previously-established linguistic message, seems untenable and should not serve as the basis for evolutionary models of language and speech. While there are clearly non-prosodic means of conveying emotion and intention in speech, such as through word selection and syntactic constructions, these do not circumvent the need to be externalized through a prosodic-scaffold mechanism during vocal communication.

Before moving on to present my evolutionary model of a prosodic precursor to speech and music, I will summarize the model briefly so as to facilitate the presentation (which is outlined in **Figure 6** below, as described in the section “Bifurcation to form language and music”). I will argue that there was not one but two shared stages that served as joint precursors in the evolution of language and music: (1) the first was a system of affective prosody, and (2) the second was a system of intonational prosody. In other words, affective and intonational prosodies evolved through a sequential process as two linked evolutionary stages. In addition, while the first stage was made up of innate calls, the second capitalized on the newly-evolved capacity for vocal learning in humans. Following this second joint stage, language and music branched off as reciprocal specializations, each one retaining certain key features of their joint precursor stages. The model attempts to account for modern-day similarities between music and language/speech by loading the precursor stages with as many shared features as is theoretically justified. I argued in Brown (2000a) that, given that language and music possess both shared and distinct features, it would be most parsimonious to propose that their shared features evolved first, and that their domain-specific features evolved later as part of a branching process (see also Mithen, 2005), making language and music homologous functions (Brown, 2001). This idea would stand in contrast to models contending that music evolved from speech (Spencer, 1857), that speech evolved from music (Darwin, 1871; Jespersen, 1922; Fitch, 2010), or that music and language's similarities arose independently by convergent evolution.

Before proceeding to describe the model, I want to point out that, given the absence of any clear definition of music, I am going to adopt a view of music (and singing) that leans heavily on the side of pitch and most especially on the use of scaled pitches, even if there is imprecision in the scale degrees and/or their execution by a voice or instrument (Nikolsky, 2015). As a result, I am going to distinguish music from both speech prosody and emotive vocalizations. In addition, while rhythm is a critical feature of music, there is no aspect of music's rhythmic system that is not potentially shared with either dance or metric forms of speech, like poetry. Hence, if the development of an evolutionary model of music requires that we identify domain specificity for music, then I see tonality as the principal distinguishing feature of music (see Savage et al., 2015 for ethnographic support for this). While I am familiar with myriad examples of musics that fail this definition—e.g., they are based on unpitched percussion sounds, they are pitched but are not based on fixed scales, they are more concerned with timbral changes than pitch pitches, they contain emotive vocalizations, prosodic speech, and/or whispers—I cannot see the utility of developing an evolutionary account of music based on either non-tonal or metrical features. Instead, my model posits that a non-tonal prosodic precursor was the source for the tonal system that came to characterize much music as we know it.

**Figure 3:**
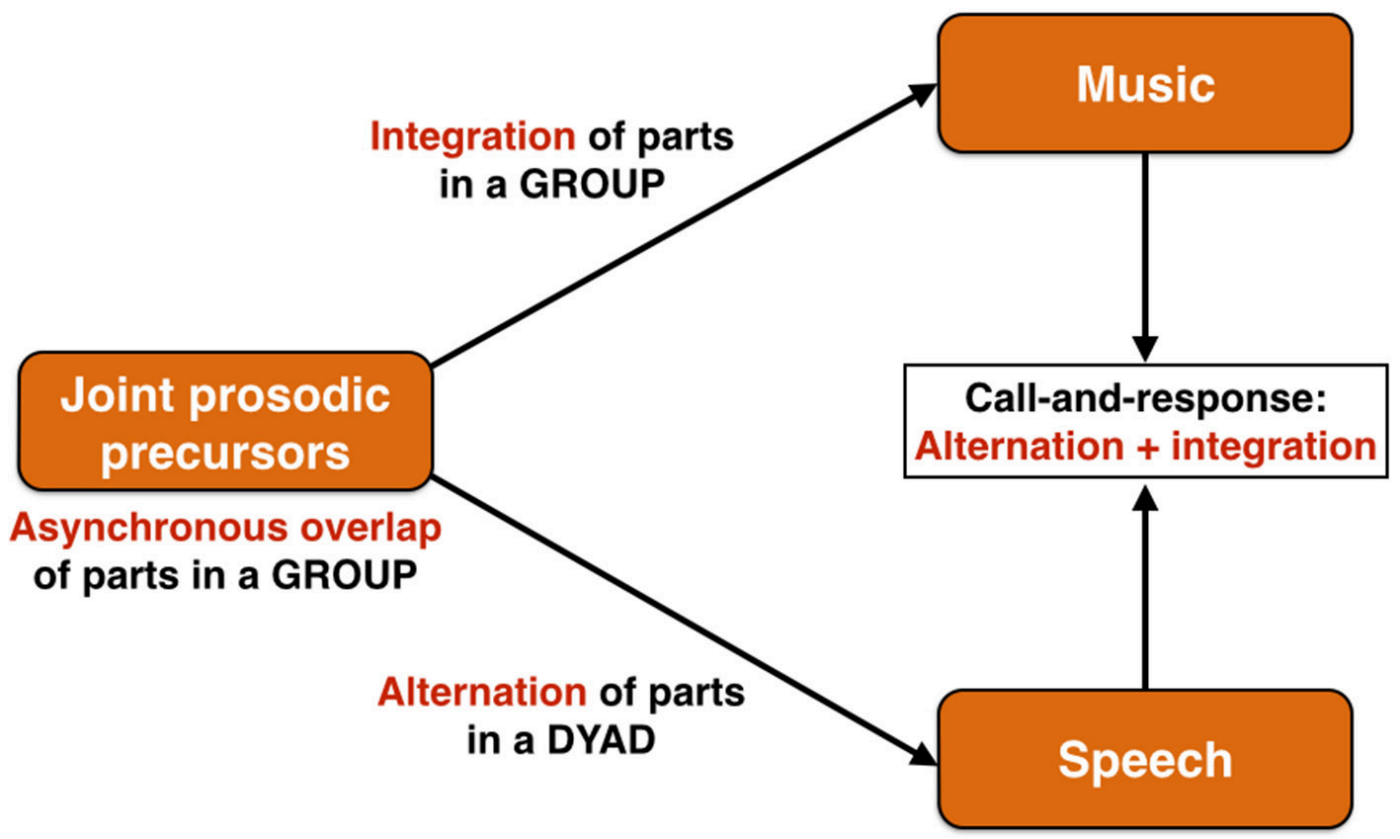
The performance arrangements of communication. The joint prosodic precursor is proposed to be a group communication system based on both affective and intonational prosodies and that is characterized by an asynchronous overlapping of parts among the individual callers. An evolutionary branching process occurs such that music would evolve to achieve a tight temporal integration of parts in a group, whereas speech would evolve to achieve an alternation of parts in a dyad, as occurs in standard conversational exchange. These distinctive communicative arrangements of music and speech are joined together in call-and-response singing, where the call part is generally performed by a soloist in alternation with a chorus. It is essentially an alternation between an individual and a group, thereby showing a combined profile of music's and speech's distinctive performance arrangements.

The first evolutionary stage of the prosodic model of language origin is proposed to be a system of affective calling derived from the mechanisms of emotional vocalizing found in mammals more generally (Briefer, 2012). I have argued previously (Brown, 2007) that, not only was this particular affective system a joint precursor for language and music, but that it was a *group* communication system, particularly one that operated in ritualized contexts, such as territory maintenance, and that acquired its group force though emotional contagion (see also Hagen and Bryant, 2003). Using a wolf chorus as a model of such a precursor, I argued that this evolutionary stage was characterized by an imprecise overlapping of parts among the group members, showing little to no synchronization of parts. This performance arrangement is referred to musically as *heterophony*^1^, which is when “different parts are performing the same melody at the same time but with different versions” (Malm, 1996 p.15). Starting from this common, asynchronous precursor, an evolutionary branching process would occur to create two different forms of coordination during communication such that (1) music would evolve to achieve a tight temporal *integration* of parts through the evolution of the capacities for both rhythmic entrainment and vocal imitation, and that (2) speech would evolve to achieve an *alternation* of parts, as occurs in standard dialogic exchange (Figure 3). This functional and structural bifurcation reflects the fact that music retains the precursor's primary function as a device for group-wide coordination of emotional expression, most likely for territorial purposes, while language evolves as a system for dyadic information exchange in which an alternation of parts, rather than simultaneity, becomes the most efficient means of carrying out this exchange. These distinctive communicative arrangements of music and speech come together in a performance style that is found quite widely across world cultures, namely *call-and-response* singing (Figure 3), where the call part is informational and is textually complex (typically performed by a soloist, as in speech) and the response part is integrated and textually simple (typically performed by a chorus, as in music). Call-and-response is an alternating (turn-taking) exchange, but one between an individual and a group, rather than two individuals.

The idea that speech evolved from a group-wide communication system has a distinct advantage over individualist accounts of language origin in that can provide a solution to the “parity” problem (Arbib, 2012). The evolution of communication systems is constrained by the fact that meanings have to be mutually interpretable in order to be adopted by a community of users. Any person can devise a word to mean bee, but unless everyone in the community understands it, then it is useless as anything more than an unintelligible device for self-expression. A group-communication system obviates this problem, since it is produced collectively. In addition, and following along the lines of the wolf example, making the group-communication system something ritualized helps in achieving meaning through the use of context specificity and the signaling of consequentiality. Communication will occur in situations that have shared emotional meanings and shared consequences for all members of the group, such as during territory defense. Having language be group-level from the start provides a solution to a number of evolutionary obstacles to achieving parity in communication.

## “Musilanguage” as a joint prosodic precursor

This first precursor stage of group-affective vocalizations that I have just described would be a ritualized territorial chorus of emotional communication. It would be neither speech-like nor music-like in its acoustic features, but instead something similar, functionally and structurally, to a non-human form of group chorusing, like a wolf chorus or a pant hoot chorus in chimpanzees. While speech and music do indeed have shared mechanisms of emotional expression (affective prosody), the affective precursor just described is lacking in many additional features that are shared between speech and music and that should be reasonably found in a joint evolutionary precursor. Hence, my model requires the existence of a second joint precursor-stage before the bifurcation occurred to generate language and music as distinct and reciprocal specializations emanating from it. While the first stage focused on the shared features of affective prosody, this second stage should now contain the shared features of *intonational* prosody that are found in speech and music.

My characterization of this second precursor stage will comprise a revised and corrected account of what I called the “musilanguage” system in a previous publication (Brown, 2000a) and which was fleshed out in book form by Mithen (2005). Hence, it will comprise my Musilanguage 2.0 model. The core idea of the model is that *those features of language and music that are shared evolved before their domain-specific features did* due to the presence of a joint precursor—what I call the musilanguage system—that contained those shared features. While the initial joint precursor described above would be a system of affective prosody, this second stage would achieve the next important level of intonational prosody. In other words, *it would embody those features of intonation that are shared between language and music*, but without requiring lexicality, syntax, or musical scale structure (tonality), an idea also developed by Fitch (2010). If I had to summarize the properties of this stage, I would argue that it is a “grammelot,” in other words a system of nonsense vocalizing or pure prosody, as was prominent as a tool for traveling theater companies in the days of the Commedia dell'Arte during the Renaissance (Jaffe-Berg, 2001; Rudlin, 2015). The only modification that I would propose is that the musilanguage precursor was a *group-level* grammelot, produced through chorusing. In what follows, I will outline a number of key properties of the proposed joint prosodic precursor, with an eye toward defining those features that can be thought of as shared between speech and music and hence that can be most reasonably attributed to a joint evolutionary stage. Table 1 lists a dozen such features.

**Table 1:** Features of the musilanguage system.

1	Vocal production learning
2	Breath phrases
3	Level tones and level transitions
4	Imprecise levels-and-contours pitch system (tonicity), with melodic motion based on pitch proximity
5	Phonemic combinatoriality (but as meaningless vocables)
6	Meaningful melodies: Holistic intonational formulas
7	Phrase structure uniting combinatorial and holistic processes
8	Affective prosody (inherited from the first precursor stage)
9	2- and 3-unit stress groupings (headedness)
10	Heterometric rhythms
11	Repetitive form
12	Polyphonic and heterophonic performance arrangements

### Voluntary control of vocalization and vocal learning

I contend that the transition from the first affective stage to this second stage corresponds to the transition from the non-human-primate system of involuntary control of stereotyped calls to the appearance in humans of both voluntary control over the vocal apparatus and vocal production learning. Belyk and Brown (2017) proposed a co-evolutionary account for the joint appearance of these two capacities, although other theorists have suggested sequential models (Ackermann et al., 2014). Hence, the advent of the musilanguage stage would mark a transition from innate to learned vocalizing, accompanied by the complexification of communication sounds that learning makes possible. This similarity between speech and music as learned vocal-communication systems is an extremely important one to keep in mind. *It places the emergence of vocal learning firmly upstream of the separation between language and music in human evolution*.

### Breath phrases

Phrase structure in both speech and music approximates the length of a breath phrase (Pickett, 1999). This may seem like a trivial similarity between speech and music as communication systems, but it also makes them natural partners when it comes to setting words to music (discussed below). There were significant changes in the voluntary control of respiration during human evolution (MacLarnon and Hewitt, 1999, 2004; Belyk and Brown, 2017), and it would seem that such changes impacted speech and music in comparable manners to influence the structural features of phrases in both domains. When people take breaths while either speaking or singing, they tend to do so at phrase boundaries, rather than in the middle of a phrase (Grosjean and Collins, 1979). In addition, the depth and duration of an inhalation correlate with the length of a produced sentence (Fuchs et al., 2013). Finally, extensive work on the analysis of pause duration as a function of the length and/or syntactic complexity of sentences points to a role of respiratory planning in speech production (Krivokapic, 2012). Hence, there is clear motor planning for speech at the level of respiration. Provine (2017) proposed that the nature of human breathing, and thus vocalization, may be a direct product of the transition to bipedal locomotion.

### Level tones and level transitions

An important feature of human vocalization that is virtually never mentioned in evolutionary accounts of speech or music is the fact that humans can produce level tones when vocalizing. Much of primate vocalizing is based on pitch glides, as can be heard in the pant hoot of chimpanzees and the great call of gibbons. While such glides are still present in human emotional vocalizations, such as in cries, both speech and music are based on transitions between relatively discrete tones. These tones are generally longer and more stable in music than they are in speech, but level tones seem to be present in speech to a large extent (Mertens, 2004; Patel, 2008; Chow and Brown, submitted). The defining feature of much music is not only that the transitions are level but that they are *scaled* and *recurrent* as well. Hence, instead of having an imprecise sequence of tones, the tones become digitized to create a small set of pitches that are used recurrently across the notes of a melody, in both ascent and descent. When this does not occur, we get a melodic pattern that is speech-like (Chow and Brown, submitted), although such a pitch pattern sounds increasingly music-like the more it is tandemly repeated (Deutsch et al., 2011).

### Levels-and-contours (L&C)

Building on the last point, a related acoustic feature of the musilanguage system is that it would be based on an imprecise (non-recurrent) and coarse-grained mechanism of pitch signaling that involved a basic sense of both pitch levels (e.g., high vs. low) and pitch contours (e.g., rising vs. falling). Importantly, this system would be *pitched but not tonal*. In other words, it would not be based on the scaled pitches that are found in the majority of musical systems, and would thus not be, in my view, a true form of singing. This idea is a modification of an incorrect proposal that I made in the original publication based on a limited database at the time about the pitch properties of speech (Brown, 2000a), about which Fitch (2010) was quite justified in raising objections. Instead, I now see the precursor system as having an imprecise relative-pitch system based on optimizing the contrast between relatively high/rising and relatively low/falling pitches, what I will refer to as a levels-and-contours (L&C) system. In twentieth century British theorizing about intonation, the term “tonicity” was used to characterize this type of pitch system (Halliday, 1967, 1970), where different types of pitch contours are used to signal intonational meaning. A key acoustic feature of this system that is shared between speech and music is that melodic movement tends to be based on pitch proximity, rather than large leaps (Huron, 2006; Patel, 2008; Chow and Brown, submitted). I will propose below that, after the separation of language and music, *speech retained the imprecise levels-and-contours system of the precursor*, while music increased the precision of the pitch relationships by introducing tonality through scale structure, making the pitches recurrent in the formation of melodies and thereby making music into a combinatorial system for pitch. Hence, the coarse-grained pitch production mechanism of the levels-and-contours system of the musilanguage stage provides a reasonable joint precursor for the pitch properties of both speech and music.

### Phonemic combinatoriality

**Figure 4:**
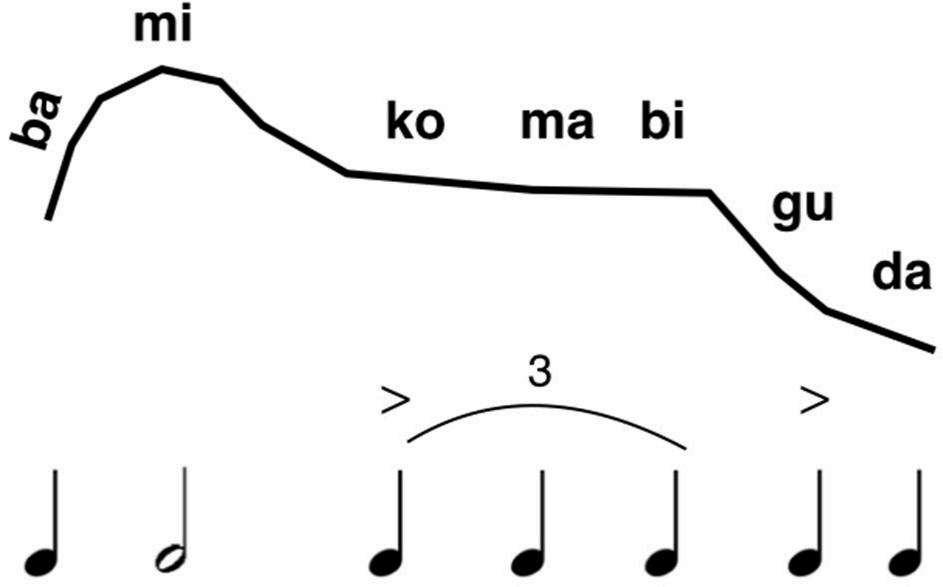
A speculative proposal for a musilinguistic phrase. The figure presents a speculative example of what a musilinguistic phrase might be like. The top row indicates the relative-pitch profile of the melody, while the bottom row indicates the relative duration values of the syllables. Phonetically, the system is comprised of a repertoire of open syllables based on phonemic combinatoriality. Open syllables are shown here, with the pairing between consonant and vowel being based on MacNeilage's (1998) model of speech evolution.

One of my major contentions is that the evolution of phonemic combinatoriality is a feature that should be placed upstream of the divide between speech and music, comprising a key property of the joint musilanguage precursor (see **Figure 6** below). This conforms with Fitch's (2010) claim that proto-language was a system of “bare phonology.” Tallerman (2013) takes issue with the concept of “phonology” being applied to anything other than meaningful words and thus true language, although I would point out that proto-language models do not present any kind of specification of the phonetic properties of their proto-words (Bickerton, 1995; Jackendoff, 1999). Hence, there was most likely a proto-phonology in place before language evolved. I mentioned MacNeilage's (1998) frame/content theory above, which seems to be as good a model as any for the origin of syllable structure through phonemic combinatoriality. Most mandibular oscillations in non-human primates are voiceless, and so a critical feature of the MacNeilage model is that the open vocal-tract configuration of the oscillation should become phonated, making syllables into pitch-bearing units. As per the point raised in the previous two paragraphs, this should permit the formation of level tones as well as glides. As such, this would favor the use of open syllables at this stage, so as to maximize information due to pitch variation. Importantly, phonemic combinatoriality would provide one mechanism for creating phrase structure by the musilanguage system, such that the vocalic part of the syllable would serve as the locus of melodic and rhythmic variation. I could imagine that the musilangauge system of proto-phonology was comprised of a repertoire of such syllabic units (see Figure 4). Given that this stage preceded the evolution of lexicality, then these syllables were *vocables*, or nonsense syllables, in keeping with the musilanguage's status as a grammelot. As in many forms of birdsong, there could have been a large diversity of such units, even though each unit would be devoid of intrinsic meaning (Marler, 2000; Slater, 2000).

### Meaningful melodies: holistic intonational formulas

Beyond the localist mechanism underlying phonemic combinatoriality, there would be a more global and holistic system of pragmatic intonational melodies that had categorical meanings (Fernald, 1989; Papousek, 1996), just as they do in modern speech. The most basic contrast would be between 1) phrases with descending contours that end in low tones, as in typical declaratives, conveying a sense of certainty, stability, and/or finality, and 2) phrases that proceed and/or end in high tones, conveying a sense of uncertainty, continuity, suspense, or incredulity (Halliday, 1967, 1970). The latter are perhaps the first questions of human communication (Jordania, 2006), conveyed via intonation alone through a grammelot, much the way that filtered speech retains the intonational meanings of sentences (Fernald, 1989). It would be hard to estimate how many melodies would exist in the repertoire of this system, but these would be pragmatically-distinct melodies that operated more or less in a categorical manner. Hence, this could be a first step in achieving phrase-level meanings and prosodic scaffolds before lexicality was introduced (Fitch, 2010), in which case the system would be better characterized as one of pure *prosody* than pure phonology. The resultant phrases could be thought of as “holophrases.” However, these would not be the *symbolic* holophrases discussed by people like Wray (1998), Mithen (2005), and Arbib (2012), but instead *prosodic* holophrases that conveyed affective and pragmatic meanings in a holistic manner, much the way that speech prosody often does. This relates to some models of speech and/or music evolution that posit a central role for mother/infant communication (Dissanayake, 2000; Falk, 2009).

### Affective prosody

By inheriting the innate expressive mechanisms from the first evolutionary stage—something that itself is phylogenetically derived from primate communication—the musilanguage system would have additional expressive modulation of phrases according to the valence and intensity of the communicated emotion, providing yet another influence on the melody and rhythm of the phrases. This occurs with regard to global and local changes in the pitch (register), loudness, and tempo of the phrases. I called these “expressive phrasing” mechanisms in the original publication (Brown, 2000a) and now see the initial affective precursor as being a specialized version of affective prosody occurring as a group territorial chorus.

### Stress groups

The system should show similarities to features of stress timing seen in speech and music, whereby syllabic units often occur in 2- or 3-unit groupings, with a sense of stress on the initial syllable (Brown et al., 2017). This conveys what linguists call “headedness” (Jackendoff, 2011), which is a hierarchical differentiation of the elements within a grouping, where emphasis is generally placed on the first element. These groupings can themselves be organized hierarchically and can potentially be embedded in one another in a recursive fashion (Jackendoff, 2011; Tallerman, 2015). The musilanguage system is thus proposed to have hierarchical phrase organization, an important feature shared between music and speech (Lerdahl and Jackendoff, 1983; Lerdahl, 2001).

### Heterometric rhythms

The rhythmic properties of this system would not be the isometric type of rhythm found in much music, but instead the “heterometric” type of rhythm that is characteristic of speech (Brown et al., 2017). Instead of having a single, fixed meter, the rhythm might involve changes in stress patterns, but still maintaining the primacy of 2- and 3-unit groupings and patterns.

### Repetitive form

To the extent that such a communication system would be both ritualized and performed in groups, it might have a strongly repetitive type of form (a so-called “ostinato” form), as in much music in indigenous cultures (Lomax, 1968) and beyond (Margulis, 2013, 2014). Hence, the same phrases would be uttered repeatedly. Figure 4 presents a highly speculative account of what a musilinguistic phrase might look like in terms of (1) phonemic combinations to diversify the number of syllable types, (2) the predominant use of open syllables, (3) the overall melodic contour of an arching intonational formula (as one example of such a formula), and 4) the local grouping-structure of the rhythmic units, but with a non-metric rhythm overall. Such a phrase might be uttered repeatedly by a given individual during group chorusing. Compared to the vocalizations of the first evolutionary stage, this would be a learning-based system that permitted voluntary control of vocalizing, although still occurring in a ritualized manner at the group level.

### Polyphonic texture

**Figure 5:**
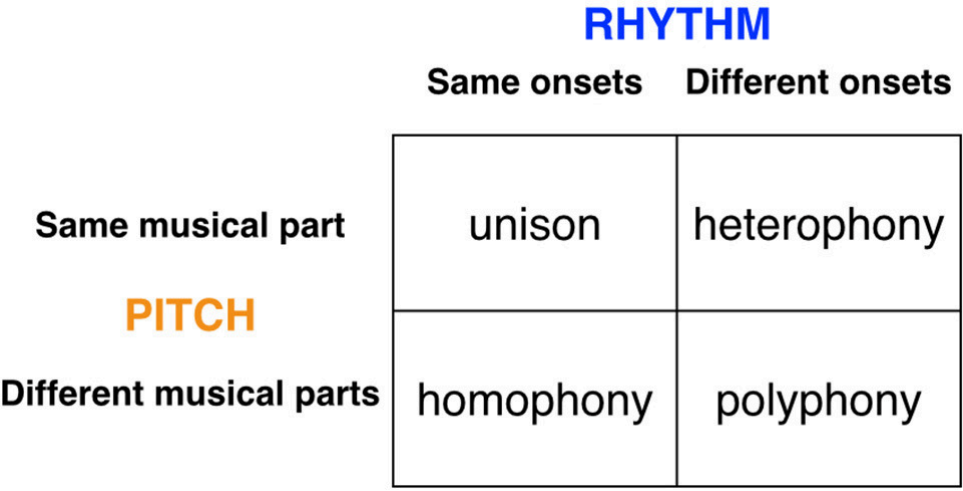
Textures of chorusing. The figure presents an overview of the major types of textures (i.e., multi-part performance arrangements) found in chorusing, both animal and human. The figure is organized as a 2 × 2 scheme, where one dimension relates to pitch (whether the melodic lines are the same or not) and the other relates to rhythm (whether the various parts are synchronized in time or not). Each cell indicates a principal type of choral texture. The right-side cells are found in both animals and humans, while the left-side cells are principally found in humans. It is proposed that the initial affective precursor of language and music was heterophonic, while the musilanguage stage was potentially polyphonic as well. The aligned textures of unison and homophony required the emergence of the human capacity for metric entrainment in order to evolve.

Finally, in order to think about the performance arrangement of the musilanguage system, Figure 5 presents an overview of the major types of “textures” (i.e., multi-part performance arrangements) found in both human and animal chorusing. The figure is organized as a 2 × 2 scheme, where one dimension relates to pitch (whether the melodic lines are the same or not) and the other dimension relates to rhythm (whether the various parts are either synchronized in time or not). I argued in Brown (2007) that the initial evolutionary stage of group-level affective prosody was characterized by a “heterophonic” texture in which each individual of the group performed a similar melodic line but in which the parts were asynchronous in onset, as seen in a wolf chorus (see also Figure 3 above). There are many examples of such chorusing in animals and humans (Filippi, 2016). In order to make the musilanguage stage more language-relevant, I would argue that, in addition to the presence of heterophony, this system would show the new texture of *polyphony*. Polyphony allows for two significant changes in the structural properties of performance compared to a heterophonic system: (1) there is a diversification of the vocal parts and hence the possibility of differentiation according to communicative roles, and (2) there is some degree of alternation of parts. The musilanguage stage would start to show some signs of alternation, which is a defining feature of conversation and a key feature of call-and-response musical forms. This is a first step toward having a differentiation of parts, both in terms of content and presentation, hence permitting a leader/follower dynamic. However, instead of having the seamless separation of parts that occurs in conversation, this stage would most likely have an imprecise type of exchange, in which the alternating parts overlapped with one another, as seen in a number of primate and avian duets, for example in gibbons and duetting birds (Dahlin and Benedict, 2013). One implication of the proposal that I am making here is that the capacity for vocal learning arose before the capacity for rhythmic entrainment and integration (contra the proposal of Patel, 2014). Hence, the musilanguage system, while voluntary and learned, would still have a relatively poor capacity for the synchronization of parts. I will return to this important point in the next section about the evolutionary changes that made music possible.

What would be the function of the musilanguage system as a group-level grammelot compared to the first stage of innate affective expression? In keeping with my attempt to optimize the shared prosodic features of language and music before their separation, I would say that the system could be involved in group communication but in functions more dyadic as well. For example, a simple call-and-response interaction might be a novel arrangement of this system, showing some basic capacity for the alternation of parts and thus the roots of the information exchange that occurs in dialogue. While the syllabic units would be meaningless, the intonational melodies might be able to be used referentially to convey emotional meanings about objects in the environment or the actions of others, hence communicating consequentiality in a non-lexical and prosodic fashion.

## Bifurcation to form language and music

**Figure 6:**
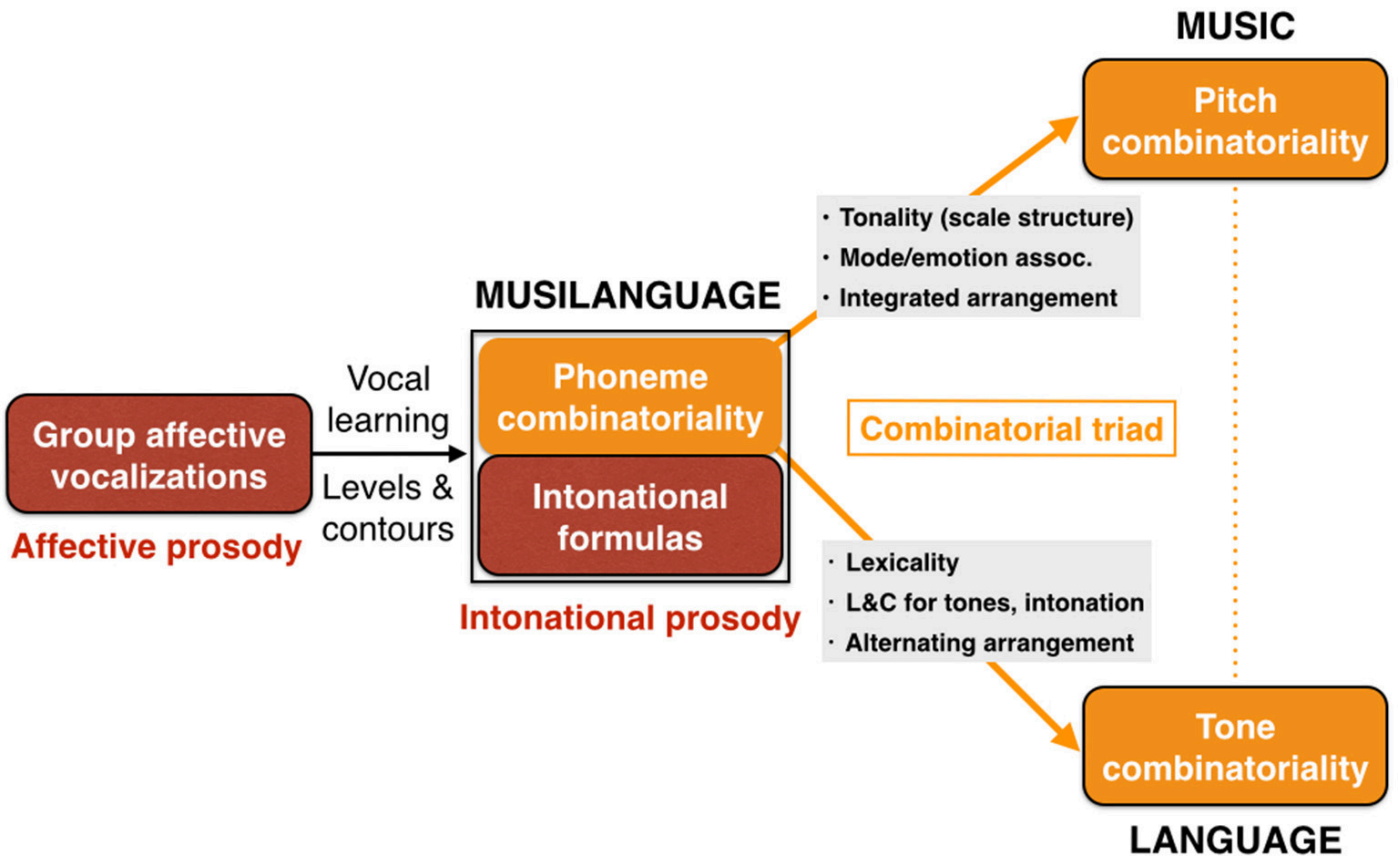
The Musilanguage 2.0 model. Three evolutionary stages are shown. Stage 1 at left is one of group emotional vocalizations based on innate calls driven by the mechanisms of affective prosody. Stage 2 in the middle is the musilanguage stage of intonational prosody, possessing the various features outlined in Table 1, including vocal learning and the levels-and-contours pitch system. Two of its key features are highlighted here, first a system of phonemic combinatoriality (where orange in the figure signifies combinatorial), and second a system of holistic intonational formulas (where dark red signifies prosodic and holistic). Stage 3 at right is the bifurcation to form music and language as separate, though homologous, functions. The road to music involves a digitization of the pitch properties of the levels-and-contours precursor to develop tonality based on scale structure. This is accompanied by a system of emotional-valence coding that I call “scale/emotion” associations. The performance arrangement is integrated due to evolutionary changes permitting entrainment using metric rhythms. A domain-specific combinatorial feature of music is pitch combinatoriality. Next, the road to language involves the capacity to generate words through acoustic symbols. Externalization of language through speech is proposed to retain the levels-and-contours (LandC) system used by the musilanguage precursor. I propose that speech evolved as a lexical-tonal system from its inception, one that worked based on combinatorial principles. Language develops a performance arrangement that is based on alternation. In orange is presented a “combinatorial triad” of phonemic combinatoriality (shared between music and language), pitch combinatoriality (specific to music), and tone combinatoriality (specific to language).

With this description in mind of two sequential precursor-stages shared by language and music, we can now examine the bifurcation process to form full-fledged language and music as distinct, though homologous, functions, as well as their (re)unification in the form of songs with words, including call-and-response chorusing. Figure 6 presents an overview of the model, starting with the innate group calling of affective prosody, followed by the musilanguage system of intonational prosody. The figure highlights the important proposal that phonemic combinatoriality is a shared feature of language and music, and this forms a critical part of what will be jointly carried over during the bifurcation process. I will first talk about language (lower part of figure) and then move on to discuss music (upper part of the figure).

### Language

In thinking about the birth of lexicality in acoustic symbols, I am going to propose that we consider two unconventional though long-established ideas, namely sound symbolism and lexical tone, as well as their union through a “frequency code” in which lexical tones could operate in a sound-symbolic manner (Ohala, 1984, 1994). I find it reasonable to consider the notion of sound symbolism as a potential origin of symbols, a timeworn idea that dates back to the ancient Greeks. Just as gestural theories of language origin are predicated on the idea that gestural pantomimes were the road to achieving gestural symbols (Armstrong and Wilcox, 2007; Arbib, 2012), *so too acoustic pantomimes could have been the road to achieving acoustic symbols* (Figure 1). While much research on sound symbolism focuses on phonemic effects related to vowels and consonants (e.g., front vowels connoting small size vs. back vowels connoting large size), a small amount of research relates to what Ohala (1984, 1994) referred to as a “frequency code,” in which pitch could be used to iconically convey symbolic meanings. Such a pitch-based code serves as the foundation non-symbolically for affective communication in many animal species and in infant-directed speech, but also has a limited potential to iconically encode spatial features of objects. For example, Nygaard et al. (2009) demonstrated that pitch was effective as a cue to perceive not only emotional valence in speech, but also size and temperature. Perlman et al. (2015) showed that participants could modulate pitch in non-linguistic vocalizations to convey information about vertical position, speed, and texture. Interestingly, sound symbolism has been found to apply to *lexical tone* as well (Ohala, 1984, 1994), with high tones being associated with words conveying small size, and low tones being associated with words conveying large size (so-called size symbolism). While there is no question that arbitrariness ultimately came to dominate the lexicon, it seems reasonable to hypothesize that language evolution got its start by capitalizing on the processing advantages that iconicity could offer (Kita, 2008; Perniss et al., 2010; Imai and Kita, 2011; Perlman and Cain, 2014).

A second hypothesis that I would like to present is that spoken language evolved as a system of lexical tones from its inception (cf Jespersen, 1922), rather than tone being a late emergence. My original model (Brown, 2000a) mistakenly argued that the musilanguage precursor had the property of lexical tone and thus lexicality, an objection well pointed out by Fitch (2010). I now firmly reject that idea in favor of lexical tone being a purely linguistic feature that emerged *after* the separation of language from the musilanguage precursor. From a cross-linguistic perspective, we know that the majority of spoken languages in the world today are lexical-tonal, although they are concentrated into a handful of geographic hotspots, mainly sub-Saharan Africa, southeast Asia, Papua New Guinea, and the Americas (Yip, 2000). These languages, despite their absence in the well-studied Indo-European language family, represent the dominant mode by which people communicate through speech. Non-tonal languages are the exception, not the rule.

In proposing that language started out as a lexical-tonal system from its origins, I am claiming that the vocal route for developing acoustic symbols involved not just a combinatorial mechanism for phonemes but *a combinatorial mechanism for lexical tones* as well (Figure 6). While lexical tone is not conceived of as a combinatorial system by linguists, it seems reasonable to me to think about it this way. Each tone language has a discrete inventory of lexical tones, either level tones (e.g., high, low), contour tones (e.g., rising, falling), or some combination of the two (Yip, 2000). The majority of syllables receive one of these possible tones. Importantly, tone languages contain a large number of homonyms that vary only in tone but in which the phonemes are identical; the four tonal homonyms of /pa/ in Mandarin are a well-cited example of this, where the four words mean eight, to pull out, to hold, and father, respectively (Lee and Zee, 2003). Hence, lexical tone seems to operate similar to the phonemic combinatorial mechanism, but instead works on the pitch levels and/or pitch contours of the vocalic part of the syllable. In other words, while phonemic combinatoriality is principally an articulatory phenomenon, tone combinatoriality is mainly phonatory. An important feature of this hypothesis is that speech inherited and maintained the imprecise levels-and-contours melodic system of the musilanguage system. Lexical tone operates using general pitch contours with imprecise pitch targets (most commonly rising and falling) and likewise with level tones having equally imprecise pitch targets (most commonly high and low). It is critical to keep in mind that, given that lexical tone is absent in one third of contemporary languages, tone is clearly a dispensable feature of a language. However, according to the hypothesis I am offering here, lexical tone is the ancestral state of spoken language, and the loss of tone is a derived feature of non-tonal languages, rather than the reverse progression (Brown, 2000a).

The last feature about the road to language indicated in the lower part of Figure 6 is the emergence of alternating textures associated with dyadic exchange (see also Figure 3). Given that language is about communicating information symbolically, alternation is a much more efficient means of effecting this transmission than simultaneous production, in contrast to music, where simultaneous production is central to its coordinative function and efficacy. I will return to this point about alternation in the section below about the evolution of syntax, since recent work on interactional linguistics demonstrates not only the prosodic relatedness of interacting speakers (Couper-Kuhlen, 2014; Bögels and Torreira, 2015; Filippi, 2016; Levinson, 2016) but their syntactic relatedness as well, leading to models of “dialogic syntax” (Du Bois, 2014; Auer, 2015).

### Music

The road to music is characterized by a complementary set of features emerging from the joint musilanguage precursor. The imprecise nature of the pitch-targets for lexical tone is contrasted with their precision in music and its system of tonality using scaled pitch-levels, which comprises the second major branching from the musilanguage system (Figure 6). As paradoxical as it might sound, speech's lexical tones are not the least bit tonal in the musical sense, although both lexical tone and music operate using relative pitch-changes, rather than absolute pitches. Tonality in the musical sense involves a discrete inventory of (relative) pitches making up a musical scale, thereby establishing fixed interval-classes among these pitches, where the same pitches are generally used in both melodic ascent and descent, what I refer to as the *recurrence* of pitches.

Importantly, music is a third type of combinatorial system in human vocal communication (beyond phonemic combinatoriality and lexical-tonal combinatoriality), however in this case involving specific pitch combinations, similar to certain forms of melodious birdsong (Marler, 2000). As mentioned above, while phonemic combinatoriality focuses mainly on articulation, music's pitch combinatoriality focuses mainly on phonation, as with lexical tone. What makes music “musical,” and what makes it acoustically different from lexical tone, is that the pitches are scaled and recurrent, whereas in speech, whether for a tone language or an intonation language, they are not. In addition, this scaling of pitch occurs both in the horizontal dimension of melody and in the vertical dimension of harmony (another manifestation of recurrence), since music retains the complex group textures of the precursor stages, although the evolution of rhythmic entrainment mechanisms provides music with a wide diversity of texture types, including human-specific forms of unison and homophony (see Figure 5 above).

I propose that the coarse-grained levels-and-contours system that was ancestral to speech and music, and that was retained by speech after the bifurcation process, ultimately gave rise to the musical type of tonality, by making a shift from the imprecise pitch-targets of the precursor to the precise intervallic pitch-targets of music. The road to music occurred by a digitization of the pitch properties of the prosodic precursor to produce a scaling of pitches, which serves as the basis for tonality and thus music. For example, as pointed out by the early comparative musicologists (Sachs, 1943), there are simple chants in indigenous cultures that alternate between only two pitches. So, I can imagine scenarios in which the imprecise system of the precursor became quantized so as to settle on recurrent pitch targets through scaling principles.

The final point about the road to music that is indicated in the upper part of Figure 6 is the emergence of integrated textures associated with group-wide production (see also Figure 3). As shown in Figure 5, the most integrated textures in human communication are unison and homophony, due to the joint onset of parts. These are the most domain-specific and species-specific textures of music, compared to both human conversation and animal forms of group vocalizing, where heterophony and polyphony predominate. The emergence of integrated forms of chorusing is due to the advent of mechanisms of not just vocal imitation but metric entrainment (Brown, 2007). While entrainment is often discussed in the literature as the synchronization of movement to some external timekeeper (as in a person tapping their finger to a metronome beat), it occurs comparably as *mutual* entrainment among individuals engaged in chorusing or related forms of synchronized body movement, like marching (Chauvigné et al., 2014).

**Figure 7:**
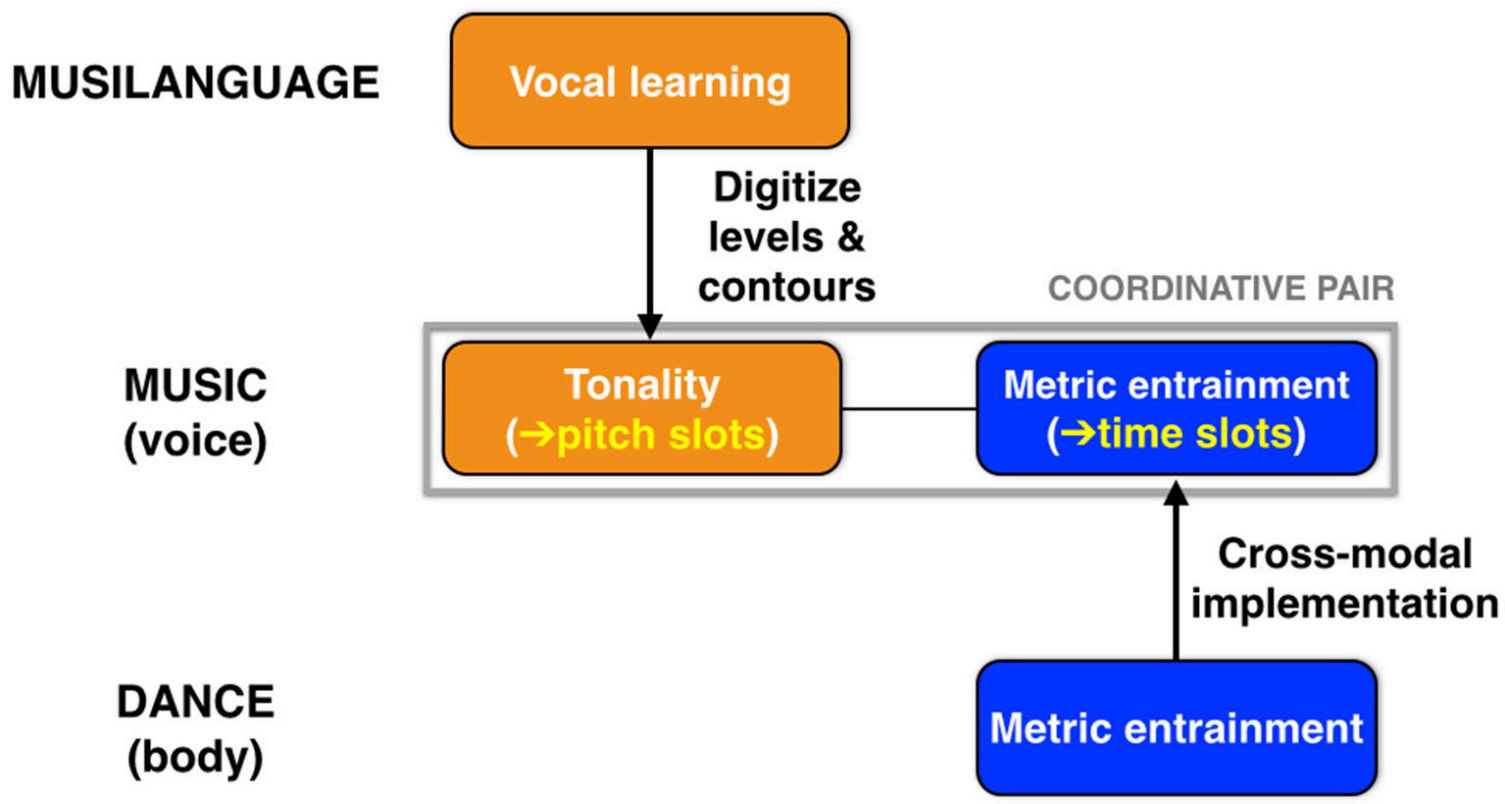
Music as a dual coordination system. Music evolved to be a dual coordination system, using both tonality and metric entrainment to engender integration. Scale structure (tonality) provides “pitch slots” for coordination, while metric structure provides “time slots” for coordination. As shown at the top, music inherited vocal learning from the musilangauge precursor, and achieved scale structure through a digitization of the levels-and-contours pitch system of that stage. Metrical structure is proposed to be a cross-modal system that originated as mutual entrainment of whole-body movement during group dancing. Music is able to co-opt this cross-modal system as its rhythmic mechanism.

A major hypothesis of this article is that music evolved to be a *dual* coordination system, using both tonality and metric entrainment to engender integration (Figure 7). Scale structure, by digitizing the occurrence of usable pitches, creates *pitch slots* for coordination among chorusing individuals, as manifested in the vertical grid-like pattern of a musical staff, with its discrete pitch levels. Likewise, metrical structure, by creating discrete beat locations for onsets, creates *time slots* for coordination among individuals. The extreme case of integration in music occurs in unison texture—as in the group singing of “Happy Birthday”—where all performers converge on the same pitches at the same time points. However, while music is indeed a dual coordination system, I contend that *scale structure is music's defining feature*, with metrical structure being something that is shared with dance and even with speech in the cases of poetic verse and the rhythmic chanting that occurs at political rallies. In support of this, it is clear that tonality and metrical structure can each work in isolation, as seen both in non-metrical melodies and in metrical structures that are unpitched, such as a tap dance.

An important evolutionary question is how tonality and meter came together to create the dual coordination system that we associate with music. I am inclined to think that entrainment evolved primarily in the context of whole-body synchronization through *dance* (Brown et al., 2006a; Brown and Parsons, 2008; Chauvigné et al., 2014), and that musical chorusing later co-opted this whole-body entrainment system to create musical integration. This jibes with the fact that tonality is domain-specific but that metrical structure is used in a cross-domain fashion, being multi-effector (voice and body) and multi-sensory (we do not need pitch at all for metrical structure or entrainment). Hence, the integrated nature of music emerges from the marriage of a domain-specific pitch system of tonality and a domain-general timing system. Patel (2014) has argued that the trait of metric entrainment evolved jointly with vocal learning, and that the two are casually related to one another. However, I do not agree with that perspective. Since vocal learning is a shared feature between music and speech, I believe it should be placed at the level of the joint vocal precursor described above. In contrast, I see entrainment as emerging outside of this vocal nexus as a system for whole-body coordination through dance, which later gets co-opted by the musical system for use in vocal chorusing and its instrumental analogs (Figure 7). To my mind, the relevant co-evolutionary question is not that between entrainment and vocal learning (as per Patel), but instead that between entrainment and *tonality* to create music's dual coordination system.

Before concluding this discussion about the evolution of music, I would like to point out that the evolutionary mystery of music is not just the generation of scale structure *per se*, but the cognitive perception that different scale-types have different emotional valence connotations (Huron, 2006, 2008; Bowling et al., 2012; Parncutt, 2014). I will refer to this as “scale/emotion associations.” In Western music theory, there is an association between the major scale and positive emotional valence, and between the minor scale and negative emotional valence. Scale types can be used in a contrastive manner by composers in a narrative context to convey different emotional meanings, much as contrastive facial expressions can be used by actors to convey different emotions to audiences.

**Figure 8:**
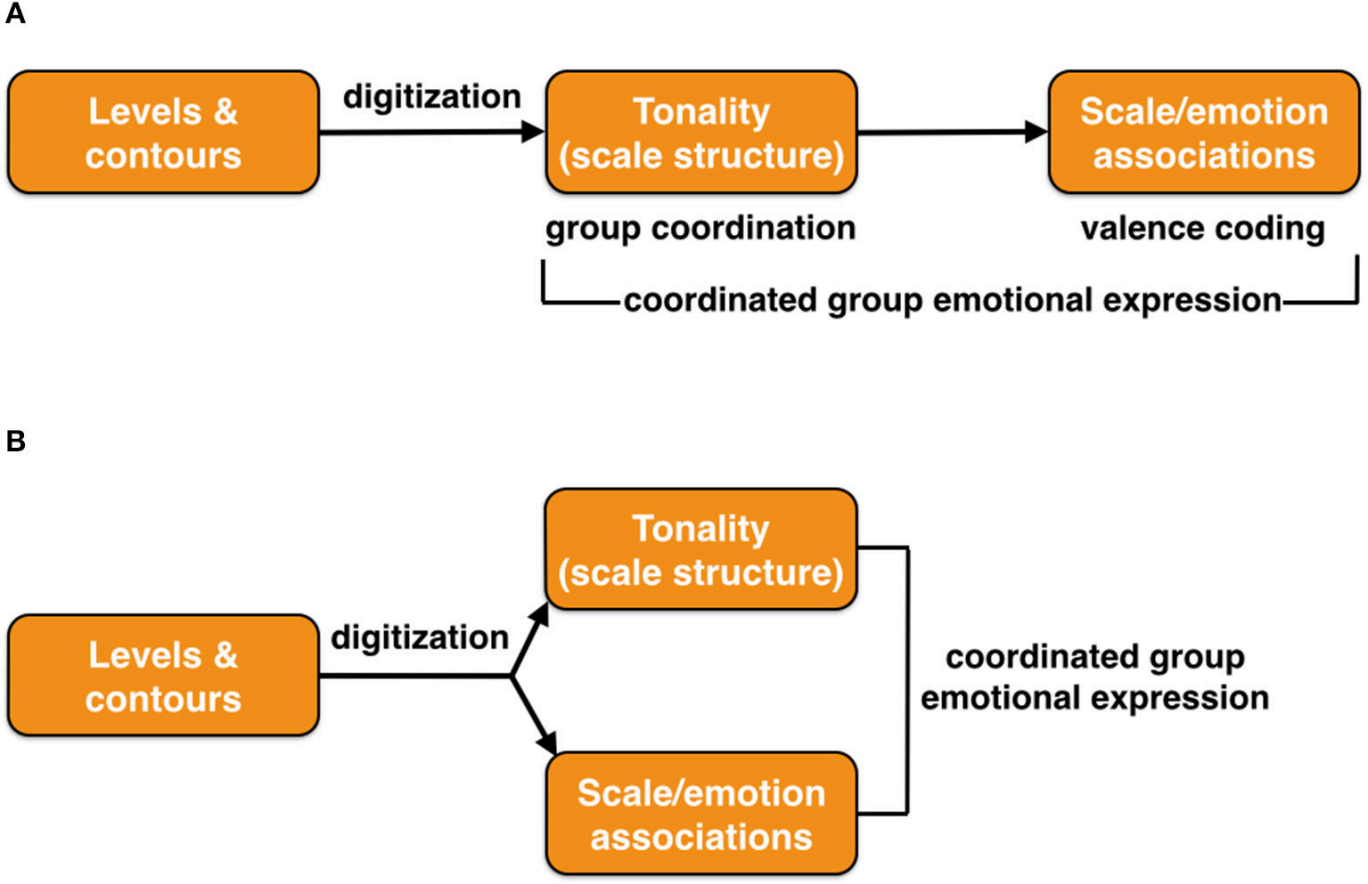
The origin of scale/emotion associations in music. Two models are presented for how scale/emotion associations may have evolved, either **(A)** as a sequential occurrence, or **(B)** as a joint occurrence. In both cases, the ultimate outcome is coordinated group emotional expression. The figure is meant to represent an evolutionary timeline, with time progressing from left to right.

Did scale structure and scale/emotion associations evolve as a unitary phenomenon or instead as two sequential emergences? I could imagine scale structure as serving a coordinative function for group integration all on its own, separate from valence coding, for example as in a musical version of a wolf chorus. Scale/emotion signaling would be more important for emotional expression, by creating a musical language of emotion based on valence coding, involving the contrastive use of two or more scale types. This would be important for group-wide emotional communication. Figure 8 compares two possible evolutionary models for the emergence of scale structure and scale/emotion processing.

The end result of the musilanguage precursor and its branching to form speech and music is the emergence in humans of a “combinatorial triad” (Figure 6) comprised of (1) phonemic combinatoriality for both speech and music, derived from the musilanguage precursor, (2) lexical-tone combinatoriality specific to tone-speech, and (3) pitch combinatoriality specific to music. These systems routinely come together, combining the phonemic and pitch domains. Common examples are the singing of songs with words, such as “Happy Birthday,” in which music's tonal properties are combined with speech syllables to musicalize these syllables (discussed in more detail in the last section). But even in the case of singing using vocables (like la-la-la), which is predominant in many world cultures (Lomax, 1968), all singing has to occur using some phoneme or another as the articulatory vehicle, even if it is just a single vowel (as in chanting on /a/) or a nasal consonant (as in humming), although I think that Fitch's (2010) claim that music is bare phonology misses the critical point about tonality.

## Syntax evolution and the “prosodic scaffold”

In the introductory section, I argued against a strictly syllabic interpretation of the origin of speech and instead suggested that we need to put emotion, prosody, and communicative intent front and center in our evolutionary thinking, leading me to propose a “prosodic scaffold” perspective. This is the idea that the production of speech is *embedded in prosody*, rather than prosody being an add-on to the compositional and combinatorial levels of speech after the symbolic level of sentence formation has been completed. Importantly, prosody transcends the level of the individual speaker, influencing the process of alternation that characterizes speech's performative arrangement (Robinson, 2005). Recent work on both interactional linguistics and interactional prosody demonstrates the profound influence of this interaction on what people think and say (Couper-Kuhlen, 2014; Du Bois, 2014; Auer, 2015). Speech is not just a process of communication but a process of *coordination*, and prosody serves as both a cause and an effect of this coordination.

**Figure 9:**
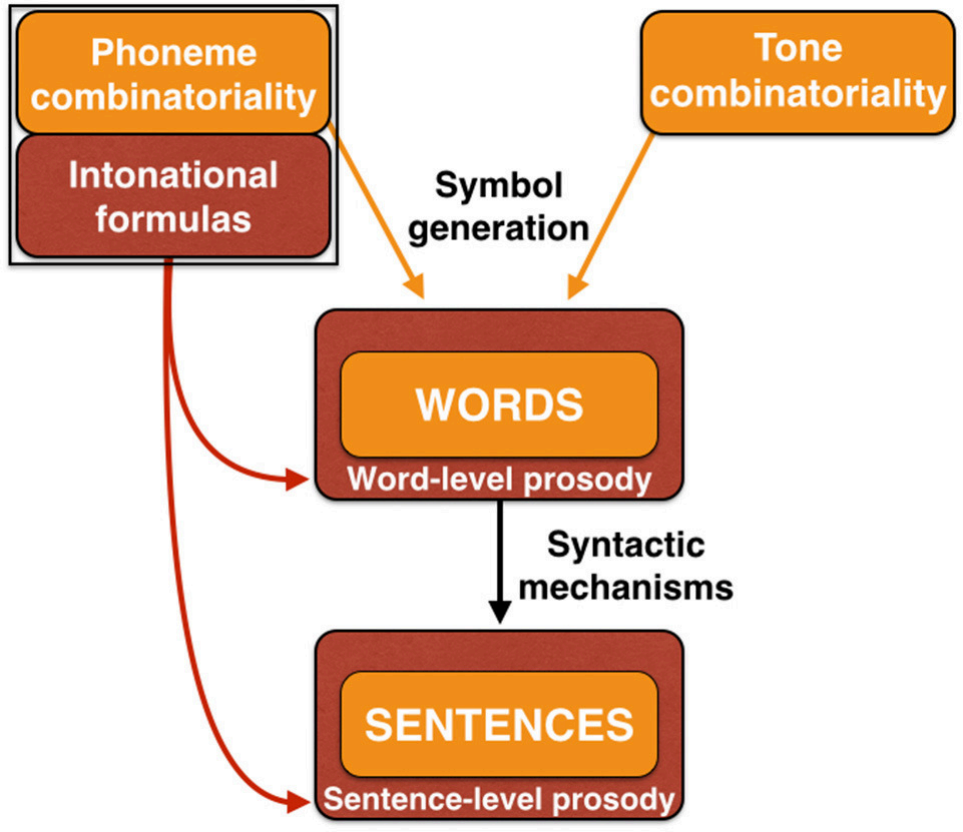
A prosodic scaffold in language generation. This figure reproduces the language-relevant material from Figure 6, using the same color-coding scheme. Phoneme-combinatorial mechanisms in concert with symbolic-meaning systems generate words as acoustic symbols. In a majority of languages, this is accompanied by the use of lexical tone as well. The “prosodic scafford” model suggests that words are embedded in word-level prosody (dark red color). Sentences are formed through compositional syntactic mechanisms. This process is proposed to be embedded in prosody, in this case sentence-level prosody derived from intonational formulas and affective prosody.

Figure 9 is an expansion of the material shown in Figure 6 but which now adds the symbolic components of words and sentences. (For ease of interpretation, material from Figure 6 unrelated to speech is removed). The prosodic scaffold is graphically represented by showing words and sentences (orange color) embedded in prosody (dark red color). At the lowest level of the linguistic hierarchy, the acoustic symbols that comprise individual words are embedded in the context of *word-level prosody*. This would occur through a modulation of the pitch, loudness, duration, and timbral features of constituent syllables to convey both linguistic prosody (e.g., lexical tone, the relative stress of syllables in polysyllabic words) and affective prosody (the valence and intensity of the communicated emotions).

The combination of words to form phrases and sentences brings us to the domain of syntax, without question the most contentious issue in the study of language evolution (Berwick, 2011; Tallerman, 2013). In the previous section, I talked about a “combinatorial triad” for the phonological aspects of speech and music. Syntax too is based on a combinatorial mechanism, but one that is quite different from the ones for phonemes, syllables, lexical tones, and pitches. In this case, it is words that get combined to form sentences, a process of combinatoriality that is referred to as *compositionality* (also productivity). Compared to the small pool of phonemes that go into the combinatorial systems for phonology, the compositional system operates with a pool of tens of thousands of words in the lexicon, organized into word classes that get combined through rule-based syntactic operations to achieve a meaningful ordering of words (Tallerman, 2015).

The field of syntax evolution has witnessed an interesting debate between two contrasting perspectives. The first is the idea of compositionality rooted in the concatenation of symbols, exemplified by “proto-language” models of the type of Bickerton's (1995). The core idea is that, starting from a basic lexicon of individual symbols, these symbols can be combined to form more-complex meanings. At the proto-language stage, the ordering of these symbols is merely associational, and does not suggest any kind of temporal ordering of events or causal relationships, although Jackendoff (1999, 2002) has suggested that Agent First might be a mechanism operative at the proto-language stage. Later stages in the evolution of syntax are thought to add grammaticalization onto the proto-language system to develop word classes that have syntactic functions, not just semantic meanings. Hence, word order and morphology develop, both of which affect the combinability of words as well as their ability to be displaced within sentences. An alternative theory is that speech began from its inception as holistic utterances, called holophrases, and that the evolution of syntax proceeded by fractionating these holophrases into words and phrases (Wray, 1998; Mithen, 2005; Arbib, 2012). The idea is that holophases conveyed complex but holistic meanings, which could be later broken down into constituent words by decomposing their complex meanings. Hence, language evolution proceeded from the holistic level to the unit level, rather than the reverse.

A critical discussion of evolutionary syntax models is beyond the scope of this article. The only point that I will add to the debate is that the “prosodic scaffold” model has the potential to synthesize elements of the two aforementioned classes of theories. The model presented in Figure 9 integrates combinatorial and holistic processes through the mechanism of prosodic embedding, as shown by “sentence-level prosody” in the figure. Prosody operates in a holistic fashion and is thus inherently holophrastic. The proposal of a prosodic scaffold is that the compositional mechanisms of syntax are *embedded in this holistic prosodic scaffold*. Therefore, instead of arguing for the idea of *symbolic* holophrases, I am arguing for the existence of *prosodic* holophrases that serve as the scaffold for compositional syntactic mechanisms.

Note that this proposal of prosodic embedding is precisely *opposite* to the way that most linguists think about language and its origin. Tallerman (2013:476) states: “Put simply, in syntax words must come first; phrases are built up around single words…Thus, suggesting that phrases evolved in protolanguage *before* there were word classes is once again entirely the wrong way round” (emphasis in the original). This is a difficult argument to address since structural linguists do not consider prosody to be a core component of language. The dispute between linguists and people like me, Mithen and Fitch might have far less to do with our proposals of a co-evolutionary stage uniting language and music as with *how prosodic processes are situated with respect to the core linguistic processes of semantics, syntax, and phonology*. If prosody is linguistically ancillary, then there is no point in discussing a prosodic proto-language that preceded semantics and syntax. If it is primary, then it makes sense to do so. I do not believe that the fields of either linguistics or language evolution have actually had a discussion on this topic.

Leaving aside the idea that language is primarily a vehicle of thought—such as in the monologue that makes up inner speech—language is routinely generated in a discursive manner though the alternating performance arrangement of speech. Sentences must therefore be generated in an interactive manner. But it would be wrong to think of a dialogue as simply a pair of monologues punctuated by interruptions. Sentences are generated during conversation *in response to* what has been said by others (Auer, 2015), not least through the exchange of questions and answers (Jordania, 2006). Language production during conversation, therefore, is a balancing act between two competing needs. On the one hand is the “monologic” driving force of leadership that aims to get one's personal information and perspective across to other people through persuasion, including statements of demands, commands, suggestions, desires, opinions, values, norms, etc. On the other hand is a “dialogic” driving force of mutuality that fosters exchange by *adapting to* one's conversation partner through an ongoing alternation between follower and leader roles. A huge literature on the pragmatics of language (Robinson, 2005) indicates the great extent to which people modify all aspects of linguistic and paralinguistic production so as to adapt to their conversational partners. This occurs at the levels of topic, word choice, syntax, pitch, loudness, tempo, and beyond. The end result of this mutual adaptation is that there is a strong sense of matching, mirroring, and mimicry between conversational partners (Couper-Kuhlen, 2014), impacting not only language and speech but posture, facial expression, gesture, as well as all aspects of prosody (Szczepek Reed, 2012, 2013; Couper-Kuhlen, 2014; Auer, 2015). Hence, the scaffold of one speaker is clearly influenced by that of another in constructing sentences during conversation.

## (Re)unification: the musilinguistic continuum

A previous section described the bifurcation process to generate language and music as separate, though homologous, functions emanating from a joint prosodic precursor that I called the musilanguage system. As a last step, I now need to consider the (re)unification of language and music (Brown, 2000a), which occurs ubiquitously in the performing arts and religious rituals. The most general interaction between language and music is unquestionably *songs with words*. The potential for direct and seamless coupling between musical pitches and the syllables of words is one of the strongest pieces of evidence for a joint origin of music and language.

However, this coupling does not occur in a singular manner. The comparative musicologist Curt Sachs presciently argued that there was not a unique origin of music but instead multiple (Sachs, 1943). In particular, he proposed a distinction between (1) a type of music derived from melody (“melogenic”) and (2) a type of music derived from words and text (“logogenic”). Quite separate from evolutionary considerations *per se*, we can think about Sachs' distinction from a purely structural standpoint and define the melogenic style of singing—whether it occurs with or without words—as being the conventional version of music using scaled intervals and metrically-organized beats. While most forms of melogenic singing in Western culture use words, many others in world cultures do not use words, but instead use vocables (i.e., meaningless syllables like “la” or “heya”) as the syllabic vehicles for vocal production.

In contrast to this melogenic style, there are many forms of word-based singing that sound like stylized versions of speech. The focal point of communication is the text, and melody is a means of accentuating it emotively. This logogenic style of singing words is basically a chanting of speech in which the melody and rhythm are closer to speech's intrinsic melody and rhythm than to the melogenic style of scaled pitches and metric rhythms. My interpretation of Sachs' multi-origins hypothesis is that the melogenic style, most notably when using vocables instead of text, arises during the divergence of music from the joint prosodic precursor, and that the logogenic style is something that follows the full-fledged emergence of speech as a cognitive function, where chanting is a means of stylizing the linguistic message.

**Figure 10:**
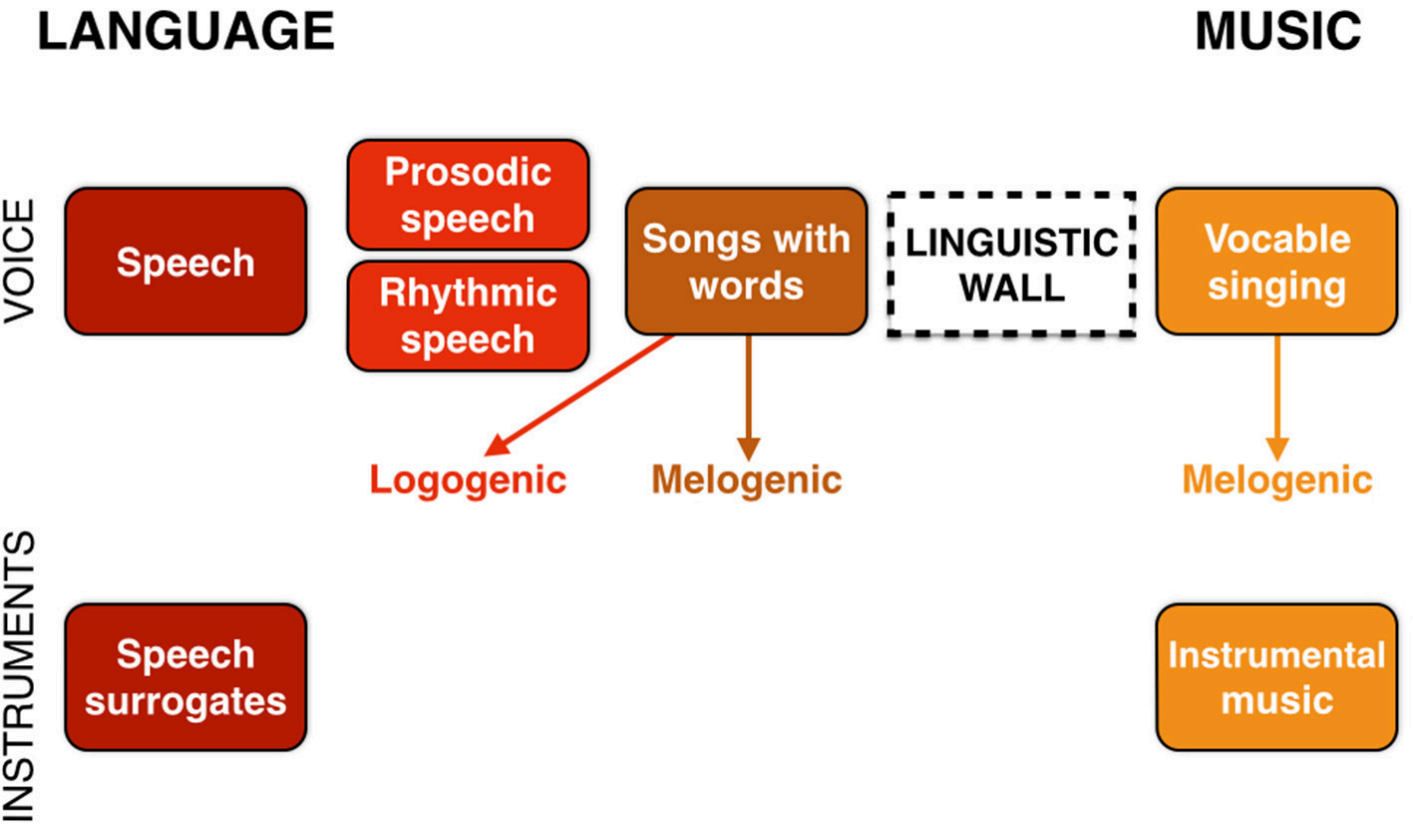
The musilingusitic contiuum. At the extremes of the continuum are language and music, represented both vocally and instrumentally. In the middle of the continuum is the most interactive function of songs with words. This can be accomplished in either a logogenic or melogenic manner. Sitting in between standard speech and songs with words are intermediate functions in which the lexicality of speech is maintained but in which the acoustic properties of the production lean in the direction of music, such as rhythmic speech and prosodic speech. There are no comparable intermediate functions on the music side of the continuum since a “linguistic wall” ensures that lexicality is a categorical feature, rather than a continuous acoustic feature like musicality. Vocable singing is shown here as being in the melogenic style. It need not be, but is most commonly found in this form in world musics.

The argument just described leads me to propose, as I did in Brown (2000a), that the evolutionary processes that generated language and music as reciprocal specializations from a joint precursor ultimately resulted in a “musilinguistic continuum” containing the poles of language and music as well as a number of interactive and intermediate functions (see also Savage et al., 2012). Figure 10 presents a model of this. At the extremes of the continuum are language and music, represented both vocally (as speech and vocable-based singing, respectively) and instrumentally. The latter includes speech surrogates, such as drummed and whistled languages (Stern, 1957), as well as conventional instrumental music. In the middle of the continuum is the most interactive function of songs with words, where language and music most universally come together (Savage et al., 2015). As shown in the figure, this can be accomplished in a logogenic manner that sounds like a stylized version of speaking, or it can occur in a more melogenic manner, employing musical scales and metric rhythms.

Sitting in between standard speech and songs with words are intermediate functions in which the lexicality of speech is maintained but in which the acoustic properties of the production lean in the direction of music. This can occur with respect to rhythm, melody, or both. Rhythmic speech is a common form of this (Cummins, 2009, 2013), as occurs in poetic verse, rap, and the group chanting that routinely permeates political rallies and marches. Prosodic speech includes the emotionally-accentuated speaking style of an actor, poet, or public speaker, or of a mother interacting with her baby (Fernald, 1989; Papousek, 1996). It also includes logogenic musical forms, such as *sprechstimme*, recitative, and parlando-style chanting, for example cantillation of the Torah. It is important to point out that there are meaningless forms of speech that still have normal speech-like prosodic contours. Examples include the filtered speech used in psychology experiments (Fernald, 1989), grammelots used in theatrical performance (Jaffe-Berg, 2001; Rudlin, 2015), or simply me watching a film in a foreign language. The point I want to make here is that the elimination of the lexicality of these forms of speech does not suddenly convert them into music. I reject the idea that prosody is a form of music and that prosodic contours divorced of words are a type of singing. Vocalizations like grammelots are better thought of as “de-lexicalized speech” than as music.

While it is easy to talk about intermediate forms of speech that are more or less “musical,” I contend that we cannot do the same thing on the musical side of the continuum. As shown by the white box in the figure, I argue that there is a “linguistic wall” that ensures that lexicality is a *categorical* feature, rather than a continuous acoustic feature like musicality. The right side of the figure shows the purely non-lexical functions of vocable singing and instrument playing. If the singing were now to include words instead of vocables, it would immediately jump the linguistic wall to become a song with words. It is difficult to imagine functions in which musicality is maintained but in which lexicality is intermediate between words and non-words. There is really nothing intermediate between words and non-words (e.g., vocables, pseudowords, grunts). So, the linguistic wall creates a categorical divide between vocable singing and word singing, making the musilinguistic continuum both asymmetric in structure and partially discontinuous.

This brings me full circle to the critique that I raised in the opening section of this article of models of language evolution that posit a singing-based precursor stage (Darwin, 1871; Jespersen, 1922). Some people might think that this stage should be identical to what I am calling the musilanguage system in this article. However, I do not see things that way. In particular, the musilanguage stage is proposed to *lack* both tonality and meter, since it is pre-musical. It is a grammelot, hence making it acoustically far more similar to prosodic speech than to music (i.e., it is comprised of levels-and-contours, not scaled intervals). So, while musilanguage might sit next to vocable singing in terms of its absence of lexicality, it would definitely sit next to prosodic speech in terms of its acoustic properties. That is why I find it inappropriate to refer to this precursor as “singing” and why I find it problematic that singing-based theories of language fail to make a distinction between music and prosody. To my way of thinking, tonality (scale structure) is a novel, domain-specific feature of music not shared with speech. That is why I far prefer a neologism like musilanguage to the term singing, since the musical features of singing-based precursors are not specified by people who use the term singing. What they are generally implying is a prosodic vocalization system, rather than a true musical system. I have argued that such a precursor embodies the shared prosodic features of language and music, but not the scales that are specific to music.

## Testability of the model

Table 1 above lists a dozen proposed features of the musilanguage system. Some of them represent features shared by speech and music, while others do not. For example, much research has shown that affective prosody is conveyed in a parallel manner in speech and music, capitalizing on the same types of dynamic cues (Juslin and Laukka, 2003). By contrast, experimental work from my lab has explored the potential musical properties of speech, and has found that speech is atonal (Chow and Brown, submitted) and based on heterometric rhythms (Brown et al., 2017), both of which conform with properties of the proposed precursor. Likewise, work on singing by Pfordresher and Brown (2017) suggests that music might in fact be a derivative of a coarse-grained levels-and-contours system, as shown by the highly imprecise nature of sung intervals in everyday singers, not to mention children (Welch, 1979a,b, 2006).

Regarding brain localization, the bifurcation model suggests that music and speech/language should show their greatest similarities at the sensorimotor (phonological) level (Brown et al., 2006b, 2009), but the least similarity with regard to domain-specific features like lexicality and tonality. For example, semantic areas in the inferior and middle temporal gyri are frequently activated during language tasks (Xu et al., 2009; Visser et al., 2012; Krieger-Redwood et al., 2015), but not during music tasks, although these areas can be modulated by music when the task is specifically focused on semantic properties (Koelsch et al., 2004). While the brain network for semantics has been well-studied, that for tonality has been much more poorly explored. A key objective for future research will be to examine the neural basis of what I have called scale/emotion processing, not least the emotional-valence connotations of different scale types. This will unquestionably lead to an exploration of limbic and paralimbic areas associated with emotion perception (Tabei, 2015).

Work on infant development supports the bifurcation model in that the first year of life appears to comprise a shared stage in which prosody, speech, and music are relatively undifferentiated, followed by a separation of language/speech and music/singing as distinct audiovocal functions (Papousek, 1996). Of course, parental singing to/with children in Western culture almost invariably involves the use of songs with words. As a result, most children are taught music through its coupling with language.

The aspect of the model that needs the most verification is the proposal of a “prosodic scaffold” in the production of speech. Work on speech perception demonstrates a strong influence of prosodic cues on comprehending speech (Filippi et al., 2017), but very little work of this type has occurred at the generative level. While numerous studies of affective prosody have used trained actors to convey different basic emotions in speech (Scherer, 2003), no studies have looked at this in spontaneous speech. A mood-induction procedure (Scherer, 2003; Van Dyck et al., 2013) might be one manner to address the influence of affect on speech production, especially if the content of the speech could be controlled for, say through a pre-learned text.

## Conclusions

The account of language evolution that I have presented in this article is vocal (rather than gestural), prosodic (rather than articulatory or syllabic), group-level (rather than individual, or dyadic), committed to a joint origin of language and music, and rooted in the idea that syntax-based phrase generation emerged, from its origin, as the filling out of a prosodic scaffold during speech production. I propose a two-step evolutionary process: first an involuntary but ritualized system of affective prosody, followed by a learning-based system of intonational prosody grounded in phonemic combinatoriality. From there, language and music branched out as separate, though homologous, functions through the emergence of lexicality and tonality, respectively, and through the adoption of the contrasting communicative arrangements of alternation and integration, respectively. After their separation, language and music are perennially reunited in songs with words, occurring in both melogenic (more-musical) and logogenic (more speech-like) styles. This potential for direct and seamless coupling between words and musical pitches is one of the strongest pieces of evidence supporting a joint origin of language and music.

## Author contributions

SB conceived of the ideas and wrote the manuscript.

### Conflict of interest statement

The author declares that the research was conducted in the absence of any commercial or financial relationships that could be construed as a potential conflict of interest.

## Footnotes

^1^I am using the term heterophony in this article so as to be consistent with similar ideas about the evolution of musical texture that I described in Brown (2007) in an article entitled “contagious heterophony”. However, the editor of this research topic, Aleksey Nikolsky, takes issue with my usage, and suggests that his own term “isophony” (Nikolsky, 2016, Appendix V) is a better description of the choral texture that I am alluding to. He defines isophony as a “brief call, continuously reproduced by multiple performers with irrational deviations in timing and pitch, where each participant retains idiosyncrasy of the rhythmic, timbral, and directional attributes of the pitch contour – altogether producing a ‘jumbled’ effect.” This strikes me as an excellent description of the phenomena that I am referencing here. Readers are encouraged to see Nikolsky's commentary on the current article for a more detailed description of isophony as applied to the evolutionary origins of musical/linguistic communication in humans.
